# Too few staff, too many patients: a qualitative study of the impact on obstetric care providers and on quality of care in Malawi

**DOI:** 10.1186/s12884-015-0492-5

**Published:** 2015-03-21

**Authors:** Susan Bradley, Francis Kamwendo, Effie Chipeta, Wanangwa Chimwaza, Helen de Pinho, Eilish McAuliffe

**Affiliations:** School of Health Sciences, City University London, 1 Myddelton Street, London, EC1R 1UW UK; University of Malawi, College of Medicine, Centre for Reproductive Health, Blantyre, Malawi; Heilbrunn Department of Population and Family Health, Averting Maternal Death and Disability Program (AMDD), Mailman School of Public Health, Columbia University, New York, USA; School of Nursing, Midwifery and Health Systems, University College Dublin, Dublin, Ireland

**Keywords:** Staff shortages, Workload, Mid-level cadres, Malawi, Quality of care

## Abstract

**Background:**

Shortages of staff have a significant and negative impact on maternal outcomes in low-income countries, but the impact on obstetric care providers in these contexts is less well documented. Despite the government of Malawi’s efforts to increase the number of human resources for health, maternal mortality rates remain persistently high. Health workers’ perceptions of insufficient staff or time to carry out their work can predict key variables concerning motivation and attrition, while the resulting sub-standard care and poor attitudes towards women dissuade women from facility-based delivery. Understanding the situation from the health worker perspective can inform policy options that may contribute to a better working environment for staff and improved quality of care for Malawi’s women.

**Methods:**

A qualitative research design, using critical incident interviews, was used to generate a deep and textured understanding of participants’ experiences. Eligible participants had performed at least one of the emergency obstetric care signal functions ^a^ in the previous three months and had experienced a demotivating critical incident within the same timeframe. Data were analysed using NVivo software.

**Results:**

Eighty-four interviews were conducted. Concerns about staff shortages and workload were key factors for over 40% of staff who stated their intention to leave their current post and for nearly two-thirds of the remaining health workers who were interviewed. The main themes emerging were: too few staff, too many patients; lack of clinical officers/doctors; inadequate obstetric skills; undermining performance and professionalism; and physical and psychological consequences for staff. Underlying factors were inflexible scheduling and staff allocations that made it impossible to deliver quality care.

**Conclusion:**

This study revealed the difficult circumstances under which maternity staff are operating and the professional and emotional toll this exacts. Systems failures and inadequate human resource management are key contributors to the gaps in provision of obstetric care and need to be addressed. Thoughtful strategies that match supply to demand, coupled with targeted efforts to support health workers, are necessary to mitigate the effects of working in this context and to improve the quality of obstetric care for women in Malawi.

## Background

The human resources for health (HRH) crisis in low-income contexts continues to hamper efforts to reduce maternal mortality. Shortages of staff have a significant and negative impact on maternal outcomes [1] and decrease the ability of health systems to maintain a state of ‘readiness’, where sufficient skilled staff, furnished with the supplies, equipment and resources that they need, are available and ready to respond to women 24 hours a day, 7 days a week [2]. Without these personnel, countries face an uphill battle to meet their Millennium Development Goal (MDG) 5 target to reduce the maternal mortality ratio (MMR) by two-thirds and increase the proportion of births attended by skilled health workers [3].

In Malawi the shortage of HRH is severe. The use of mid-level cadres has a long history here and includes a clinical officer cadre, which was introduced in 1976 [4], medical assistants, and nurse-midwives at registered, enrolled and technician grades. Mid-level providers, particularly nurse-midwives, provide the bulk of primary health care and form the backbone of the health service. Stiff opposition from professional bodies interrupted the production of mid-level nursing cadres in the 1990s. However, the emigration of 79% of degree level nurse-midwives trained between 1993 and 2002 [1] allowed a climate where the 3-year enrolled nurse programme could be reintroduced. In addition, the government of Malawi has been ambitious in trying to address workforce shortages and worked with the international community to launch an Emergency Human Resource Programme (EHRP) in 2004 [5]. This focused on a massive scale up of pre-service training of eleven key cadres but, as an emergency response, paid less attention to issues of quality and performance [6,7]. Substantial gains have been made, but vacancy rates still remain high and fall below the EHRP targets, with shortfalls against projected posts of 72% for clinical officers, 53% for registered nurses and 60% for enrolled/nurse-midwife technicians [8]. The situation has been described as ‘dire’ in health centres, where very few achieve the required minimum staffing complement and where availability of on-site staff drops drastically at night and weekends. Staff, particularly in the rural areas where 85% of the population live [9], continue to face challenging work environments characterised by high workloads, lack of essential resources and health technologies, and inadequate supervision or managerial support. These factors combine to severely compromise health workers’ abilities to deliver adequate quality care and have been implicated as drivers of disrespectful intrapartum care [10].

Critical shortages of skilled staff are a major bottleneck in the provision of timely and quality obstetric care [11], which has a significant impact on maternal and neonatal outcomes. Given the HRH challenges faced by the Malawi Ministry of Health and Population (MOH), it is unsurprising that the country is significantly off track to meet its MDG5 target of 155 maternal deaths per 100,000 live births by 2015. There is some interagency disagreement on the exact figures, but it is clear that Malawi’s MMR is falling. The WHO estimates a decrease between 2005 and 2013 from 570 to 510 maternal deaths per 100,000 live births [12]; while figures from within Malawi suggest a fall from 994/100,000 in 2004 to 675/100,000 in 2010 [9]. When this research was carried out in 2008 skilled attendance at birth was 54% [13] but had risen to 71% by 2010 [9], indicating that there has been a sharp increase in facility-based delivery. In 2007, the MOH took the step of prohibiting traditional birth attendants (TBAs) from conducting deliveries and banning women from using their services, with fines for non-compliance issued by community chiefs [14]. This may have had an impact in increasing facility-based delivery, but the ban was rescinded in 2010 and the role of TBAs remains unclear.

The consequences of staff shortages for emergency obstetric care (EmOC) are evident. Maternal death reviews in Malawi have shown that health worker factors are one of the major contributors to maternal death [15,16] and significant contributors to poor outcomes for labouring women. Lack of sufficient skilled staff plays a dual role in the phases of delay [17]. Firstly, delays in receiving timely and appropriate care on reaching a health facility (Phase 3 delays) can be directly caused by having too few skilled staff available to carry out adequate care [18]. This intersects with resource and equipment shortages that delay onset of appropriate treatment and leave skilled staff unable to carry out their professional role or to operate to the required standard [19]. Staff shortages may also cause delays in initiating emergency interventions [11]; delays in authorising referral or referring upwards to busy senior staff; or lack of performance of the EmOC signal functions^a^ [20]. Secondly, delays in deciding to seek care (Phase 1 delays) can be attributed to the consequences of some of the issues identified in Phase 3 delays, as poor quality care erodes community trust and confidence, and so delays the decision to seek care when complications arise [21,22].

While the consequences for women of inadequate HRH are well documented, there is less information on how this impacts on obstetric care providers in low-income countries. There is consistent evidence from high-income contexts that nurses value practice environments that ensure enough staff to provide adequate patient care [23]; a broad consensus on the relationship between job demands (such as workload, time pressure and staffing levels) and burnout, particularly emotional exhaustion [24,25]; and a positive, significant association between high-nurse patient ratios and burnout [26]. The limited evidence from sub-Saharan Africa shows the negative effect of staffing levels and workload on nursing cadres, predicting outcomes such as burnout [27], while research in Malawi has indicated that staff perceptions of insufficient staff or time to carry out their work can predict key variables concerning motivation and attrition [28], as well as resulting in sub-standard care and poor attitudes towards women [29]. These factors drive a vicious cycle where the lack of an enabling environment leads to poor quality clinical care and disrespect, which in turn dissuade women from facility-based delivery, encourage them to bypass facilities with a bad reputation, or to delay seeking care and then arrive in critical condition [16,21,30,31]. This exacerbates the challenges facing health workers in trying to assist them, further increases the risk to maternal health, and is a source of demoralisation and burnout for obstetric staff.

In order to untangle this vicious cycle it is crucial to understand the reality for health workers. This paper explores the perceptions of EmOC providers in Malawi on the critical factors of staff shortages and workload in their health facilities. The aim is to look more deeply at the impact on these cadres, rather than to just explore dissatisfaction, and to use the insights gained to suggest policy options that may contribute to a better working environment for staff and improved quality of care for Malawi’s women.

## Methods

This was a qualitative research study that took place as part of the larger *Health Systems Strengthening for Equity: The Power and Potential of Mid-Level Providers* (HSSE) project. The HSSE study aimed to expand the evidence base on effective use of mid-level health workers in emergency obstetric and neonatal care, and to identify the factors that would optimise the retention of these cadres and support their performance. Data were collected in three countries. This paper reports on qualitative findings from the Malawi data.

### Study setting and sample

The HSSE project took place in 25 of Malawi’s 28 districts. Three districts that had been part of a recent human resources study were excluded to avoid overburdening staff. The study focused on district hospitals, health facilities and health centres that were designated to provide EmOC, and included both government run and Christian Health Association of Malawi (CHAM) institutions.

Eligible participants were health workers who had performed at least one of the EmOC signal functions ^a^ in the previous three months and who had experienced a critical incident within the same timeframe. Nursing and midwifery staff were located in the maternity wards, but other cadres involved in obstetric care, such as clinical officers and medical assistants, also work in other departments. To ensure that these staff were represented the data collection team enlisted the help of departmental in-charges to identify potential participants from these cadres.

### Critical incident interviews

A qualitative critical incident methodology [32] was used to generate a deeper and more textured understanding of participants’ experiences. This methodology aims to identify salient events that have had a pivotal impact on a person’s experience of their job [33] and is valued in many fields of research. It provides a flexible tool that can explore factors that are not well understood, using cognitive, affective and behavioural accounts to identify turning points and provide direct, practical implications [34]. The focus of the interview in this study was to explore the critical factors that cause most unhappiness in the job by exploring a specific incident that caused the participant to become demotivated or even to think about leaving, and to elucidate causal factors impacting on their decision to leave or to remain in post. Participants were invited to describe an incident that had a significant impact on them. The technique uses probing questions to aid reflection and enhance recall by eliciting key details, reactions and emotional responses before, during and after the incident. Participants were asked to elaborate on the factors that had led up to the incident, how they felt about this, what was done to deal with the situation, if the concerns described were still present, and what they would do if the incident were to be repeated.

### Data collection

Data collection took place from October - December 2008 and was carried out by two clinical officers and three experienced researchers who were educated to at least Bachelor’s level. Prior to going into the field a comprehensive training programme was conducted with these research team members to outline the HSSE project and methods. A total of 84 interviews were conducted.

All critical incident interviews were anonymous and were carried out at a time and place that was convenient for participants. Each interview was recorded and transcribed verbatim by the interviewer. The majority were conducted in English, but a small number of participants were more comfortable using a common local language, Chichewa. These interviews were translated and transcribed into English by researchers who are fluent in both languages.

### Data analysis

All transcripts were imported into NVivo 8 software for analysis. The data analysis team included experienced researchers in Malawi (EC, WC) and Ireland (SB). An initial framework of key themes was developed to reflect the main research questions and existing literature. The analysis team met in Malawi for one week to carry out the initial coding, compare and validate coding, and discuss emerging themes arising from the data. Emerging themes were identified using a process of inductive and deductive coding [35]. Further coding was carried out in Malawi, with each team member coding into separate NVivo files. These were merged, shared between the full analysis team, and checked frequently. Any necessary revisions to the coding template to reflect new themes and ideas emerging from the data were discussed and agreed in team conference calls. Summary descriptions for each node were generated and agreed collectively. Higher level analysis and synthesis was carried out in whole team discussions between the analysis team and the two Principal Investigators (FK and EM), informed by the data and the existing literature.

### Ethical considerations

Ethical approval for this study was obtained from the College of Medicine Research and Ethics Committee (COMREC), Malawi; the Global Health Ethics Committee Trinity College, Dublin; and the Institutional Review Board of Columbia University, New York. Written informed consent was taken from each participant and the study objectives and procedures were described. All participants were informed of the voluntary nature of participation and their right to withdraw at any stage. Confidentiality was assured and all identifying information was anonymised with a unique identity number that was only available to members of the immediate research team.

## Results

A total of 84 health workers participated in critical incident interviews and recounted specific demotivating incidents that had occurred during the previous 3 months. The majority of staff who participated in these interviews were nursing/midwifery cadres (n = 63) and over two-thirds of the sample were female (see Table 1).

**Table 1:** **Sample demographics**

**Cadre**	**Total**	**Female**	**Male**
Nurse-Midwife Technicians (NMT)	40	35 (88%)	5 (12%)
Enrolled Nurse/Midwives (ENM)^*^	12	12 (100%)	0
Registered Nurse/Midwives (RN/M)	11	10 (91%)	1 (9%)
Clinical Officers (CO)	13	2 (15%)	10 (77%) 1 unknown
Medical Assistants (MA)	8	1 (12.5%)	7 (87.5%)
Total	84	60 (71%)	24 (29%)

^*^Training of enrolled nurse-midwives has been phased out and this cadre has been replaced with NMTs, but many ENMs are still present in the health system.

Almost 70% (n = 58) of respondents had seriously contemplated leaving their current post as a result of the critical incident that had been described. The key ‘tipping points’ that influenced this decision, including poor management, financial issues, and lack of clear criteria for promotion/upgrading, have been reported elsewhere [36]. Concerns about staff shortages and workload were key factors for over 40% of staff who stated their intention to leave their current post and for nearly two-thirds of the remaining health workers who were interviewed. The findings from both these groups are reported in this paper, as they demonstrate the pervasive negative impact of the human resources for health crisis on staff.

The main themes emerging were: too few staff, too many patients; lack of clinical officers/doctors; inadequate obstetric skills; undermining performance and professionalism; and physical and psychological consequences for staff.

### Too few staff, too many patients

A common narrative among participants who had seriously considered leaving their post was the challenge and stress of being responsible for too large a number of patients, or of facing unmanageable workloads that exceeded their capacity to cope. For example, an NMT spoke for many participants when she described a critical shortage of nurses that left maternity staffing levels at a maximum of two nurses during the day, but only one at night to cover both labour ward and postnatal ward. Another health worker who was responsible for multiple wards at night echoed the challenge staff faced. *“…in maternity there is only one nurse working during the night, and that nurse is covering postnatal ward, nursery, labour ward, ante-natal ward, plus theatre when there is a caesarean section…so that is not safe for the patients, because there can be emergencies in all wards at once.”* (NMT, 3141) Staff were very aware that maternity is not like other wards and *“…an emergency can come at any time…”* (NMT, 3144) Some staff described trying to take action to ensure safer working conditions for themselves and their patients, by calling on facility management or in-charges to help them, but were usually refused assistance. Only one senior NMT described management responding to staff requests for changes to staff rosters. *“What I did was to ask the matron to allocate two nurses on night shift and two nurses as well on day shift, because previously we used to have…sometimes three nurses on day shift and only one nurse during the night duty. So it was just too much for one nurse to manage the labour ward, postnatal, surgical patients all by herself with only one ward attendant. So what I did was to allocate two nurses on night shift and two ward attendants also for night shift, so that the others could be off duty.”* (NMT, 2021) Other staff reported situations where they were responsible for multiple patients with complex needs and no one to share the workload or decision-making.

The dilemma of staff shortages was not confined to night shifts. Participants outlined the pressures they faced to keep working because there were too few staff to provide on-call cover. A senior ENM said: *“It often happened that you were supposed to be on call for one week. Then it was possible that when you were on the call you worked the whole night at the maternity because there were a bigger number of deliveries. Then in the morning…you were expected to go back to the work willy-nilly even when you are tired…I just work because I have been forced to do so.”* (ENM, 2131) Many described a context where it was difficult to find time to rest or be allowed to take leave days to which they were entitled. Managers offered locum shifts to already tired staff to make up for shortages. *“But I said: ‘I need to rest.’ Money, yes, I need money, but rest as well, because if I don’t* [rest] *I find myself to not be productive. I will find myself sleeping in the ward while patients need my attention and at the end they will say; ‘this nurse is not helping us’.”* (NMT, 3011) Health workers who lived on or near to the hospital grounds found it hard to escape. *“Because I was staying right there on the health centre, sometimes I would see people coming to call me from my house while it was not my turn to go and work…there came patients at night and they came to call me, asking me to go and work. I said that I was not supposed to be working. I am on leave; I am supposed to start my leave.”* (NMT, 2131)

The introduction of a Service Level Agreement with CHAM to provide free maternity services was thought to have encouraged more women to attend, but staff numbers were not always raised to match the increased workload. *“We are still the same members of staff, we are only 3, but the workload, the number of patients we are receiving, has increased…The time we were charging* [for maternal care] *it was an average of maybe 20 deliveries. This time around it’s around 50–60 a month.”* (MA, 1111)

### Lack of clinical officers/doctors

Another set of concerns was related to the lack of sufficient COs (locally referred to as ‘clinicians’) or doctors to deliver emergency care. This affected both COs and nursing cadres. COs reported being under pressure to attend too many patients and facing the dilemma of trying to manage too many serious cases at once. This resulted in patient care being interrupted and tasks left incomplete, while insufficient staff was a direct contributor to unnecessary maternal deaths. One CO spoke of the difficulties of trying to prioritise whom to treat but not always getting it right. *“…we had so many cases in theatre and we were just going by priorities…”* (CO, 4052) This situation was exacerbated by shortages of theatre staff to carry out emergency procedures. *“…it was an emergency but when we were trying to hunt for other people, like theatre staff, we couldn’t find one…So it pains when you feel you can help and then you don’t help and patient dies, it is very pathetic.”* (CO, 3042) This was seen as particularly demoralising, especially in circumstances where COs knew that they could have successfully intervened if the right staff had been in place at the right time.

Nursing participants revealed their appreciation of how difficult it was to have so few COs or senior staff available and articulated their awareness of the constraints under which these cadres operated. However, shortage of these health workers also impacts on nurses. *“Sometimes we only have one intern doctor. So when there is a case we all scramble for the one intern available. So if the patient is not attended to, the blame is levelled against us nurses, whilst they know that there was only one doctor. When you go to report to the doctor you can even tell that he is tired, but you still ask him to see the patient. So sometimes when you go to report a case to him you find him performing caesarean operation and he fails to see your patient till the next day and the blame is again on you, the nurse, for the negligence…when there is shortage of staff any problems that arise you are in for it…our bosses do not back us when there is a problem, whilst they are very aware that the cause of the problem is really the staff shortage.”* (ENM, 5011)

Frustrations arose when nursing cadres tried to persuade COs to attend to their patients. “*If the clinicians are busy with something, I have to convince them that they come and do something to the patient…So when we are forcing them, to make them see this patient…they complain about you because you just don’t let them go.”* (RN, 1011) Other nurse-midwives also revealed tensions that arose between nursing cadres and COs due to perceptions of unfairness in workloads and levels of responsibility. “*…the hospital always is in the hands of nurses, 24 hours. Clinicians, they come there during days and in the night they are not even there. Nurses, we even do their job because we even admit patients, we even prescribe to patients who are coming in the night. Yet they are on call, sleeping in their houses.”* (NMT, 1042) However, COs were also challenged by the situation. *“…in the obstetric, we receive a lot of patients…maybe for example we only have one* [CO] *on call and receive about twenty patients in a day, but all are in labour…about one-third of the patients we need to do caesarean section…another third of it there will be that instruction to do episiotomy deliveries. So you see, only one clinician cannot manage to receive all these at the same time. It’s unfair…while in the other departments it’s only about taking histories and giving treatments. The rest, it’s in the hands of the nursing care, while in these departments, clinicians and midwives really need to work hand in hand.”* (CO, 1081) Others felt that COs were doing more than their share and needed more support. *“…the Ministry or the management teams in the district hospitals, they should be considering the welfare of the juniors, because otherwise people are suffering in silence…juniors, clinicians, nurses, who are really doing the job, because for us in the districts we are doing a lot of procedures, of which maybe had it been at another site we are not supposed to do them.” (CO, 2172)*

### Inadequate obstetric skills

Some respondents described the use of locum staff to cover shortages, but there were problems when these did not have the necessary skills to effectively work in maternity. A maternity ward in-charge outlined the challenges this caused. *“Sometimes they don’t have those skills to perform certain tasks like emergencies, emergency obstetric procedures. So sometimes you get frustrated because maybe a baby has died, just because somebody has failed to resuscitate that baby. Or even the mother herself has died, not necessarily because there was nothing that could have been done to save her, but because someone didn’t know what to do with the lady because he or she did not have the skills.”* She also spoke of the difficulties they face when newly qualified staff who have been trained in basic EmOC procedures (signal functions 1-7^a^) do not have the confidence to carry these out. *“…so many people who are qualified, but at the same time they are not able to handle the situations…They have undergone that training, but when they are on the ground here…they still think that performing the vacuum extraction is the work of a Registered Nurse or a Clinical Officer…if that mother could have been assisted early we could save the baby, but then we end up losing the baby, just because somebody never thought of doing that. The same thing with manual removal of placenta. There are other nurses who have learnt how to perform it, but then they don’t want to do it just because they are afraid that ‘if I do this and it goes wrong, I may be blamed.’”* (RNM, 3031)

A further challenge was the practice of rotating nursing staff into the maternity department with the expectation that they would be skilled enough to cover the diverse requirements of the different wards. One participant described the impact this had on provision of care. *“…it’s like the work is beyond my control and I am trying to call my friends from paediatric ward, male ward…it happened they were only nurse technicians. They were not midwives. So it was hard for me.”* (NMT, 3011) Other staff reported being told to rely on less skilled staff. An NMT in sole charge of 44 patients was told, *“You are having auxiliary nurses there; delegate as much as you can.”* (NMT 3144)

One suggested solution was to acknowledge that maternity is different and requires different skills. *“I would suggest maybe demarcating the wards, because we combine labour ward and postnatal…that’s why work increases. Maybe if they would have demarcated the wards making labour ward to be on its own and there should be the nurses attending to the patients in labour… and special nurses attending to patients…postnatally, I think that could have helped.”* (NMT, 1091)

### Undermining performance and professionalism

Respondents reported the clear impact of the lack of adequate, skilled staff on their performance and ability to meet professional standards and expectations. They described circumstances that forced them to take short cuts. *“You really know that you are supposed to do ABC, but because maybe you are alone at that time you cannot afford to do all those steps*.” (NMT, 4121) Another NMT eloquently described the negative impact this had on women in her care.

*“I could not give total patient care. Like for the postnatal patient, what I could manage was just to give the paracetamols, not asking how they were feeling, whether the baby is well. As a result we could even miss if those could develop neonatal sepsis, because even cord care was not being done. We could only give Panadol to every mother who has delivered and the caesarean, post-caesarean patient,* [we] *could only give the antibiotics and the like, not even checking how the wound is, because whenever you are in the postnatal they would say, ‘Nurse, please, I am delivering here, the baby is out’ and then you are running to the labour ward. So it was really affecting* [job performance]*.”* (NMT, 3011)

Other staff spoke of their worries about leaving women waiting to be seen, even in emergencies. Fear of maternal death was a common theme, with some outlining the potential ramifications for their professional standing. *“Maybe the council* [the Nurses and Midwives Council of Malawi] *can decide to take my certificate because I ‘mishandled’ but it’s not mishandling, it’s that the work is too much.”* These circumstances drove some participants to consider leaving, but they were held back by an awareness of how detrimental this would be for local women. *“…if we leave, all of us, really that means there will be disaster here…so I feel if we leave we are not doing good to this hospital…It’s unfair to the hospital and the community.”* (CO, 1081)

### Physical and psychological consequences for staff

Respondents articulated a range of negative impacts on their physical and psychological well-being. Many reported feelings of exhaustion due to excessive workloads. *“The workload was just too high for a nurse to work alone during the night, especially in maternity ward, so that time I felt bad because I was getting very tired, exhausted each and every time when I finish working.”* (NMT, 5052) Others described becoming *“bust”* or struggling to balance work and family life. “*You overwork, you go home very tired, frustrated, and probably do nothing at home, you can’t even take good care of your family just because so tired at work…So sometimes for your own health you feel aah what is it that I am gaining? Though I am helping patients but I am not doing justice to myself.”* (Nursing Officer, 3031) Some spoke of this resulting in poor concentration, while one respondent resorted to leaving early or using fake excuses to get away from the workload. Others reported feelings of disengagement, where *“I was working but the whole spirit of me was not there”* (CO, 3042), or of feeling used as *“…we are understaffed here. This understaffing makes us to work even more hours. And these more hours, we work just for free. That’s like a voluntary job.”* (NMT, 1042) There were also respondents who revealed that the workload was causing them to become demotivated. *“…maternity is a place where complications arise now and then, then when you feel like ‘I would have done this but I have failed it because I am alone, there is nobody to help me,’ so in such a way, you become demolished.”* (Senior EN, 2171)

Fear of maternal death, and the possibility of loss of the nursing/midwifery certificate as a result, had significant emotional impacts on respondents. Others felt sadness and guilt about the impact on women of staff shortages. *“…if somebody dies in my hands I will feel guilty the rest of my life…”* (NMT, 3143) The ultimate solution for many staff is to leave their post, a prospect that seems to be exacerbated by the impact of maternal death or the fear that one might happen, but which contradicted their professional ethos and commitment to work with Malawi’s mothers. One NMT who had been involved in a maternal death said, *“…no one comes all the way from her home to kill a patient. You come to save people’s lives. But the way things are, it is as if we are not working and this is discouraging me and I feel like leaving the job.”* (NMT, 4051)

## Discussion

This study investigated the negative consequences of staff shortages and workload for maternal health staff in Malawi. It supports previous research that described the challenges faced by these cadres [28,37,38] and provides some insights into the systems failures in matching supply and demand that have clear negative implications for health workers and the women in their care. Permeating the results are very real concerns from obstetric care providers about the quality of care that can be provided in the prevailing circumstances.

One of the key findings is the lack of flexibility in the current system for allocating maternity staff rosters or ensuring sufficient personnel to cope with the exigencies of obstetric care. Managers were reported to rigidly apply a policy where staffing norms, such as two nurse-midwives allocated during the day and only one on duty at night, were adhered to, with little account taken of actual workload or the unpredictable nature of EmOC provision. Even when maternity staff found themselves in critical situations, with unmanageable nurse-midwife to patient ratios and dangerous circumstances for labouring women, their pleas for assistance were often dismissed. The challenges of having too few staff and too many patients were further exacerbated by the lack of an adequate skill mix to address obstetric complications. Nurse-midwives who identified obstetric problems were obliged to chase the few COs or doctors on duty to secure care for their patients, while COs struggled with the pressure of trying to prioritise whom to treat when faced with too many serious cases at once. Other participants described working with locum staff, or nurses from other departments, who lacked the necessary skills to deal with obstetric emergencies.

The combination of shortages of staff and inadequate skill mix described by participants has serious implications for a health facility’s ability to maintain a state of readiness. It is evident that managers found it difficult to staff facilities, particularly at night when staffing ratios on maternity wards were severely reduced. In high-income contexts, lack of adequate out-of-hours care (which includes not just staff numbers, but also the skill mix and deployment of those staff) has been shown to leave women and neonates vulnerable [39]. In the Malawian context, maternal death reviews have highlighted human resources constraints and/or insufficient clinical skills as key factors that delay adequate and timely care [15,16,29,40]. These factors also have important consequences for health workers. Staff in this study felt their performance and professionalism were undermined, echoing previous research that indicates service providers are acutely aware that the EmOC they provide is of poor quality [41]. In addition, the threat of maternal or neonatal death was a key driver of demotivation and a source of considerable stress and sadness.

In common with many sub-Saharan African countries, the staffing establishments in Malawi are not linked to demand per health facility, but are allocated according to health facility type [42]. This can lead to significant inequity in workloads for staff in different facilities and, indeed, in different departments within the same facility. Although staffing establishments were increased in the wake of a functional review there are still huge shortfalls in the number of staff needed to deliver basic health services to the population [8]. This lack of sufficient health workers must be viewed in the context of concerted efforts by the Malawian MOH to encourage skilled attendance at birth, in line with its MDG commitments. Participants in this study reported significant increases in the number of deliveries, mirroring the sharp increase in facility-based delivery, from a plateau of around 50% for much of the 1990s and early 2000s, to over 70% in 2010 [9]. However, there has not been a parallel increase in the number of staff to attend these women [43]. There are questions to be asked about claiming skilled attendance at birth statistics as a success in contexts where the lack of an enabling environment leaves staff overwhelmed and some women delivering alone, despite being present in a health facility.

Increasing the number of staff is necessary, but not sufficient, to address maternal mortality and morbidity. Malawi’s efforts have also addressed the skill mix needed to deliver basic EmOC, including changes to the scope of practice, training and regulation of key cadres [44]. However, participants in this study clearly indicated that some staff were not carrying out the EmOC signal functions for which they had been trained, echoing quantitative findings reported elsewhere [20]. One reason cited was fear of professional censure or blame if mistakes were made. This points to a lack of support and supervision for these staff, an area which has been recognised by district managers as needing attention and of particular concern with the influx of newly qualified staff produced by Malawi’s EHRP [45]. It also underlines a lack of transparency in the legal protections afforded to health workers, an issue that could justifiably be considered part of the advocacy and policy remit of professional bodies, such as the Nurses and Midwives Council of Malawi. At the same time there are staff working beyond their skill level, facing unreasonable demands on their professional practice. Research in neighbouring Tanzania suggests that health workers influence each other’s practice, both good and bad [46]. This has worrying implications for the quality of obstetric care if staff are not performing signal functions that they should be providing, or are cutting corners due to staff shortages, and junior staff come to see this as ‘normal’ practice.

It is clear that a strategic approach is necessary to try to match supply and demand in health facilities. One mechanism to address this issue at the facility level is the use of a human resource management (HRM) tool, such as the Workload Indicators of Staffing Need [47]. This is designed to allow health care managers to assess the gap between current staffing levels and the levels required to meet a specific health facility’s workload. Measuring workload indicators (such as number of deliveries), listing key roles, and allocating service standards, provides data that can be used to inform recommendations on revising staffing norms according to demand, ensuring equitable staff/patient ratios and providing the appropriate skill mix, particularly in maternity. However, this type of assessment must be accompanied by flexibility and responsiveness in the HRM system to allow transfer of staff and creation of new posts. Although HRM policies and plans have been developed in Malawi, the capacity to implement workforce planning, recruitment, hiring and deployment, remains fragile [48] and deployment processes can be bureaucratic and slow.

Producing more staff may not be possible given the current resource constraints and it will be a challenge to address staffing inequalities in this context. However, it is clear from this study that action needs to be taken precisely because there are too few staff and the current situation is unsustainable. One option could be to improve allocation strategies to allow more effective performance of the staff who are already in the system. For example, managers could pool spare HRH in the district, to be allocated to facilities where shortages occurred or if staff leave needed to be covered. Introducing transparent processes to show fair distribution of on call and night shift allocations would also address some of the concerns raised by participants in this study and provide a mechanism to hold non-performers to account. At the health facility level there is scope for managers to be more thoughtful about best utilising and supporting existing human resources. Many interviewees revealed that they were exhausted, using words like ‘*demolished*’ and ‘*bust*’ to describe how they felt. Some reported being pressured into taking extra shifts to cover shortages, while others were not even allowed to take leave to which they were entitled. This is extremely unfair and has a clear impact on health worker motivation. Redesigning shifts is a simple solution that could be employed. Staff in this study reported instigating realistic changes that appeared to be successfully implemented. Indeed, consultation with staff and including them in the decision-making process would demonstrate support for health workers and the difficult situation they face. It would generate a degree of autonomy that is a key driver of health worker motivation and satisfaction, while allowing more choice and flexibility and providing more off-time can enhance recruitment and retention [49]. A further option, which was suggested by participants, could be implemented at the ward level. There was a prevailing view throughout the critical incident interviews that maternity is different to other wards and should be treated as a separate unit that needs specialised skills and adequate staffing levels.

A focus on respectful, quality care for women must consider the institutional context and the role of inflexible or unfair HRM practices on EmOC providers’ ability to deliver professional standards of care. There is growing evidence that the quality of care delivered by health workers may be related to the quality of their working life [50], yet respondents in this study faced considerable demands, overwhelming workloads and lack of support from their managers. The resulting dissatisfaction, demotivation and attrition will result in poorer care for Malawi’s women. In order to break the vicious cycle health care managers need to support and enable EmOC providers to deliver the high quality care that women deserve.

### Limitations

This qualitative study revealed the pernicious impact of staff shortages on health workers and the role this plays in causing staff to consider leaving their job. The majority of respondents were located in district or rural hospitals; in some health centres staff were too busy to participate in the critical incident interviews, something that could have introduced a potential bias in the study. The lack of quantitative data matching actual staff numbers to workload makes it difficult to ascertain whether perceptions about workload differed between health facilities with different staff to caseload ratios (although differences in total number of staff did not impact significantly on perceptions of workload) or if the situation was even more challenging in smaller health centres. The data were collected during a period when there was significant pressure within Malawi to increase facility-based delivery, combined with influxes of inexperienced new staff as a consequence of the EHRP. It is possible that these factors heightened staff perceptions of stress within the system, although previous and ongoing research in Malawi suggests that inadequate HRH is a persistent challenge for both EmOC staff and the women in their care.

## Conclusion

Despite substantial increases in the number of health staff in Malawi the MMR remains obstinately high. This study revealed the difficult circumstances under which maternity staff are operating and the professional and emotional toll this exacts. It is difficult to envisage how efforts to reduce maternal mortality and to provide timely, quality obstetric care can be effective if they rely on tired, overworked and unsupported staff to achieve results. Systems failures and inadequate HRM are key contributors to the gaps in provision of obstetric care and need to be addressed. Thoughtful strategies that match supply to demand, coupled with targeted efforts to support health workers, are necessary to mitigate the effects of working in this context and to improve the quality of obstetric care for women in Malawi.

### Endnote

^a^ Basic EmOC is comprised of seven signal functions (1–7). An additional two signal functions (8,9) indicate comprehensive EmOC: 1. Administer parenteral antibiotics; 2. Administer uterotonic drugs (e.g., parenteral oxytocin, parenteral ergometrine); 3. Administer parenteral anticonvulsants for pre-eclampsia and eclampsia (e.g., magnesium sulphate); 4. Perform manual removal of placenta; 5. Perform removal of retained products of conception (e.g., manual vacuum aspiration, dilation & curettage); 6. Perform assisted vaginal delivery (e.g., vacuum extractor); 7. Perform neonatal resuscitation (with bag and mask); 8. Perform surgery (e.g., caesarean section); 9. Perform blood transfusion.

## Abbreviations

CHAM: Christian Health Association of Malawi
CO: Clinical officer
EHRP: Emergency human resource programme
EmOC: Emergency obstetric care
EN/M: Enrolled nurse/midwife
HRH: Human resources for health
HRM: Human resource management
HSSE: Health systems strengthening for equity: the power and potential of mid-level providers project
MA: Medical assistant
MDG: Millenium development goal
MMR: Maternal mortality ratio
MOH: Ministry of health and population
NMT: Nurse-midwife technician
RN/M: Registered nurse/midwife
TBA: Traditional birth attendant

## References

[1] Gerein N, Green A, Pearson S (2006). The implications of shortages of health professionals for maternal health in sub-Saharan Africa. Reprod Health Matters.

[2] Gill Z, Bailey P, Waxman R, Smith JB (2005). A tool for assessing ‘readiness’ in emergency obstetric care: The room-by-room ‘walk-through’. Int J Gynecol Obstet.

[3] World Health Organization (2005). World Health Report 2005: Make Every Mother and Child Count.

[4] Chilopora G, Pereira C, Kamwendo F, Chimbiri A, Malunga E, Bergstrom S (2007). Postoperative outcome of caesarean sections and other major emergency obstetric surgery by clinical officers and medical officers in Malawi. Human Resour Health.

[5] Ministry of Health Malawi (2004). A Joint Programme of Work for a Health Sector Wide Approach (SWAp) 2004–2010.

[6] Rawlins BJ, Kim YM, Rozario AM, Bazant E, Rashidi T, Bandazi SN (2013). Reproductive health services in Malawi: An evaluation of a quality improvement intervention. Midwifery.

[7] Carlson C, Boivin M, Chirwa A, Chirwa S, Chitalu F, Hoare G (2008). Malawi Health SWAp Mid-Term Review.

[8] Ministry of Health Malawi UNICEF, UNFPA, World Health Organization, AMDD (2010). Malawi 2010 EmONC Needs Assessment Final Report.

[9] National Statistical Office (2010). Malawi Demographic and Health Survey 2010.

[10] Bowser D, Hill K (2010). Exploring evidence for disrespect and abuse in facility-based childbirth: report of a landscape analysis.

[11] Dogba M, Fournier P (2009). Human resources and the quality of emergency obstetric care in developing countries: a systematic review of the literature. Human Resour Health.

[12] World Health Organization UNICEF, UNFPA, The World Bank, Division TUNP (2014). Trends in Maternal Mortality: 1990 to 2013.

[13] UNFPA (2011). The State of the World’s Midwifery 2011: Delivering Health, Saving Lives.

[14] Ministry of Health Malawi (2007). Guidelines for Community Initiatives for Reproductive Health.

[15] Thorsen VC, Meguid T, Sundby J, Malata A (2014). Components of maternal healthcare delivery system contributing to maternal deaths in Malawi: a descriptive cross-sectional study. Afr J Reprod Health.

[16] Kongnyuy EJ, Mlava G, van den Broek N (2009). Facility-based maternal death review in three districts in the central region of Malawi. Womens Health Issues.

[17] Thaddeus S, Maine D (1994). Too far to walk: maternal mortality in context. Soc Sci Med.

[18] Knight HE, Self A, Kennedy SH (2013). Why are women dying when they reach hospital on time? A systematic review of the “third delay.”. PLoS One.

[19] Spector JM, Reisman J, Lipsitz S, Desai P, Gawande AA (2013). Access to essential technologies for safe childbirth: a survey of health workers in Africa and Asia. BMC Pregnancy Childbirth.

[20] Lobis S, Mbaruku G, Kamwendo F, McAuliffe E, Austin J, de Pinho H (2011). Expected to deliver: alignment of regulation, training, and actual performance of emergency obstetric care providers in Malawi and Tanzania. Int J Gynecol Obstet.

[21] Faye A, Niane M, Ba I (2011). Home birth in women who have given birth at least once in a health facility: contributory factors in a developing country. Acta Obstet Gynecol Scand.

[22] Kruk ME, Rockers PC, Mbaruku G, Paczkowski MM, Galea S (2010). Community and health system factors associated with facility delivery in rural Tanzania: a multilevel analysis. Health Policy.

[23] Aiken LH, Clarke SP, Sloane DM, Sochalski J, Silber JH (2002). Hospital nurse staffing and patient mortality, nurse burnout, and job dissatisfaction. JAMA.

[24] Opie T, Dollard MF, Lenthall S, Wakerman J, Dunn S, Knight S (2010). Levels of occupational stress in the remote area nursing workforce. Aust J Rural Health.

[25] Maslach C, Schaufeli WB, Leiter MP (2001). Job Burnout. Annu Rev Psychol.

[26] Sheward L, Hunt J, Hagen S, Macleod M, Ball J (2005). The relationship between UK hospital nurse staffing and emotional exhaustion and job dissatisfaction. J Nurs Manag.

[27] Engelbrecht MC, Bester CL, Van Den Berg H, Van Rensburg H (2008). A study of predictors and levels of burnout: the case of professional nurses in primary health care facilities in the free state. S Afr J Econ.

[28] McAuliffe E, Bowie C, Manafa O, Maseko F, MacLachlan M, Hevey D (2009). Measuring and managing the work environment of the mid-level provider–the neglected human resource. Human Resour Health.

[29] Beltman JJ, van den Akker T, Bwirire D, Korevaar A, Chidakwani R, van Lonkhuijzen L (2013). Local health workers’ perceptions of substandard care in the management of obstetric hemorrhage in rural Malawi. BMC Pregnancy Childbirth.

[30] Kruk ME, Hermosilla S, Larson E, Mbaruku GM (2014). Bypassing primary care clinics for childbirth: a cross-sectional study in the Pwani region, United Republic of Tanzania. Bull World Health Organ.

[31] Filippi VGA, Ronsmans C, Gohou V, Goufodji S, Lardi M, Sahel A (2005). Maternity wards or emergency obstetric rooms? Incidence of near-miss events in African hospitals. Acta Obstet Gynecol Scand.

[32] Flanagan JC (1954). The critical incident technique. Psychol Bull.

[33] MacLachlan M, McAuliffe E (1993). Critical incidents for psychology students in a refugee camp: implications for counselling. Couns Psychol Q.

[34] Chell E, Cassell C, Symon G (2004). Critical incident technique. Essential Guide to Qualitative Methods in Organizational Research.

[35] Green J, Thorogood N (2009). Qualitative Methods for Health Research.

[36] Chimwaza W, Chipeta E, Ngwira A, Kamwendo F, Taulo F, Bradley S (2014). What makes staff consider leaving the health service in Malawi?. Human Resour Health.

[37] Bradley S, McAuliffe E (2009). Mid-level providers in emergency obstetric and newborn health care: factors affecting their performance and retention within the Malawian health system. Human Resour Health.

[38] Manafa O, McAuliffe E, Maseko F, Bowie C, MacLachlan M, Normand C (2009). Retention of health workers in Malawi: perspectives of health workers and district management. Human Resour Health.

[39] Sandall J, Homer C, Sadler E, Rudisill C, Bourgeault I, Bewley S (2011). Staffing in Maternity Units: Getting the Right People in the Right Place at the Right Time.

[40] Vink NM, de Jonge HCC, Ter Haar R, Chizimba EM, Stekelenburg J (2013). Maternal death reviews at a rural hospital in Malawi. Int J Gynecol Obstet.

[41] Chodzaza E, Bultemeier K (2010). Service providers’ perception of the quality of emergency obsteric care provided and factors indentified which affect the provision of quality care. Malawi Med J.

[42] Ministry of Health Malawi (2013). Minimum Standards of Clinical Services for Malawi.

[43] Colbourn T, Lewycka S, Nambiar B, Anwar I, Phoya A, Mhango C (2013). Maternal mortality in Malawi, 1977–2012. BMJ Open.

[44] Ministry of Health Malawi (2005). Road Map for Accelerating the Reduction of Maternal and Neonatal Mortality and Morbidity in Malawi.

[45] Bradley S, Kamwendo F, Masanja H, de Pinho H, Waxman R, Boostrom C (2013). District health managers’ perceptions of supervision in Malawi and Tanzania. Human Resour Health.

[46] Spangler SA (2012). Assessing skilled birth attendants and emergency obstetric care in rural Tanzania: the inadequacy of using global standards and indicators to measure local realities. Reprod Health Matters.

[47] World Health Organization (2010). Workload Indicators of Staffing Need: User’s Manual.

[48] O’Neil M, Jarrah Z, Nkosi L, Collins D, Perry C, Jackson J (2010). Evaluation of Malawi’s Emergency Human Resources Programme.

[49] Zurn P, Dolea C, Stilwell B (2005). Nurse Retention and Recruitment: Developing a Motivated Workforce. ICN Issue Paper No. 4.

[50] West E (2001). Management matters: the link between hospital organisation and quality of patient care. Qual Health Care.

